# Vaccination in Multiple Sclerosis: Friend or Foe?

**DOI:** 10.3389/fimmu.2019.01883

**Published:** 2019-08-07

**Authors:** Tobias Zrzavy, Herwig Kollaritsch, Paulus S. Rommer, Nina Boxberger, Micha Loebermann, Isabella Wimmer, Alexander Winkelmann, Uwe K. Zettl

**Affiliations:** ^1^Department of Neurology, Medical University of Vienna, Vienna, Austria; ^2^Institute of Specific Prophylaxis and Tropical Medicine, Medical University of Vienna, Vienna, Austria; ^3^Department of Neurology, Neuroimmunological Section, University of Rostock, Rostock, Germany; ^4^Department of Tropical Medicine and Infectious Diseases, University of Rostock, Rostock, Germany; ^5^Department of Neurology, University of Rostock, Rostock, Germany

**Keywords:** multiple scleorsis (MS), immunology, vaccination, disease modifying therapy (DMT), vaccination immunology

## Abstract

Multiple sclerosis (MS) is a debilitating disease of the central nervous systems (CNS). Disease-modifying treatments (including immunosuppressive treatments) have shown positive effects on the disease course, but are associated with systemic consequences on the immune system and may increase the risk of infections and alter vaccine efficiency. Therefore, vaccination of MS patients is of major interest. Over the last years, vaccine hesitancy has steadily grown especially in Western countries, partly due to fear of sequelae arising from vaccination, especially neurological disorders. The interaction of vaccination and MS has been discussed for decades. In this review, we highlight the immunology of vaccination, provide a review of literature and discuss the clinical consideration of MS, vaccination and immunosuppression. In conclusion, there is consensus that MS cannot be caused by vaccines, neither by inactivated nor by live vaccines. However, particular attention should be paid to two aspects: First, in immunocompromised patients, live vaccines may lead to a stronger immune reaction with signs of the disease against which the patients have been vaccinated, albeit in weakened form. Second, protection provided by vaccination should be controlled in patients who have been vaccinated while receiving immunomodulatory or immunosuppressive treatment. In conclusion, there is evidence that systemic infections can worsen MS, thus vaccination will lower the risk of relapses by reducing the risk of infections. Therefore, vaccination should be in general recommended to MS patients.

## Introduction

Over the last years, especially in Western countries, vaccine hesitancy has steadily grown and poses an increasing health concern (1). The recent upsurge of measles in Europe is an impressive example. Anti-vaccinationists argue that possible side effects weigh out the benefits (2). Especially sequelaes such as autism, multiple sclerosis (MS) and various neurological syndromes have been emphasized by the anti-vaccination lobby (3, 4). This alarming development is even partly supported by health-care providers including some MS neurologists, who are afraid of iatrogenic deterioration of pre-existing MS. Indeed, studies linking vaccination and disease onset have been published. Although these studies were often underpowered and lacked an adequate design in order to provide evidence of the suspected link, they caught public awareness leading to a drop of public vaccination coverage rates (5, 6).

Epidemiological studies and pharmacovigilance data have repeatedly demonstrated safety for the vast majority of vaccines. Lately, a review concluded that there is no significant evidence for a causal relationship between the onset or deterioration of MS and vaccination against measles, mumps and rubella (MMR), influenza, hepatitis A, hepatitis B, human papilloma virus (HPV), diphtheria, tetanus, acellular pertussis, or meningococcal disease (7). Some studies have even indicated a decreased risk for MS and reduced disease activity in preexisting MS (8).

The aim of this review is to summarize data on vaccination and disease activity of both MS and acute disseminated encephalomyelitis (ADEM). Moreover, vaccination-induced effects on the immune system are presented and potential interactions between MS and immunizations are discussed.

## Basic Immunology of Vaccination

**Figure 1:**
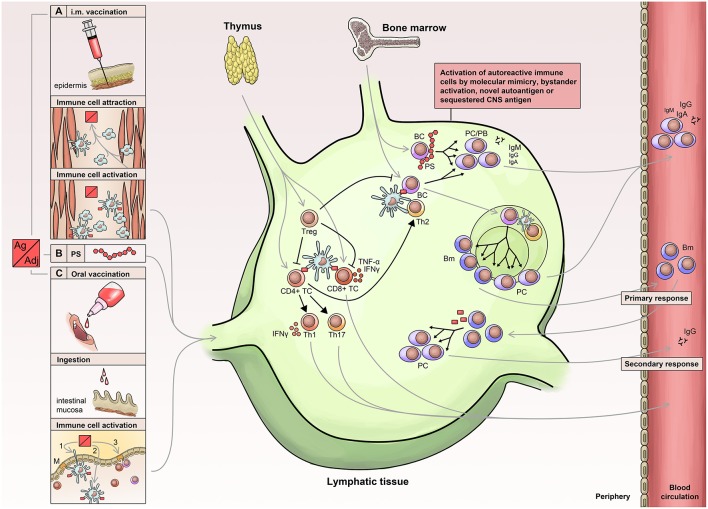
Immunology of vaccination. Routes of vaccine administration include: Injection of vaccine into muscle tissue (A) leading to attraction, activation, uptake and processing (B) in APCs (antigen-presenting cells), which then migrate to lymphatic tissue. Similarly, oral or nasal administration (C) leads to activation and migration of innate immune cells into the lymphatic tissue. APCs activate lymphocytes leading to a T cell immune response and activation of B cells, which receive additional stimuli by activated T helper cells. The primary immune response is short-lived and associated with the early appearance of low affinity antibodies, which are later replaced by high affinity antibodies generated via the germinal center reaction. PS, polysaccharide; PC, Plasma cell; PB, plasma blast; BC, B-cell; Bm, memory B cells; Treg, Regulatory T Cells.

Vaccine-induced protection is a complex issue and depends on a cascade of mechanisms and mediators (Figure 1). Eventually, protection is accomplished either by antibodies or T cell-dependent factors or by a combination of both including neutralizing or antitoxic antibodies, CD8^+^ T cells, CD4^+^ T cells and corresponding cytokines (e.g., interleukin (IL)-2, 3, 4, 5, 9, 13, 17, 21, 22, and 26) (9). Generally, vaccines have to be capable of activating antigen-presenting cells (APCs) of the innate immune system, which subsequently present the vaccine epitope(s) to T cells—the so-called ‘immunogenic potential’ (10). In this context, dendritic cells play a pivotal role due to their enhanced capability to stimulate naïve T cells (11).

The nature of vaccine-induced immunity depends on several parameters, of which the biological properties of the vaccine's epitope are of high importance (9). Live vaccines are attenuated variants of pathogens that still can activate APCs, especially immature dendritic cells, patrolling through the body. This immunogenic potential is often lost by subcellular- or subunit-based vaccines (12), which is why these inactivated vaccine antigens are usually combined with so-called adjuvants to increase and modulate the vaccine's immunogenicity via a longer lasting and more effective activation of immune cells.

One of the most widely used adjuvants are aluminum salts, which were originally thought to create a long-lasting depot of the antigen in order to provide its slow release, but have instead been shown to act on dendritic cells via PRRs (pattern recognition receptors) leading to the secretion of pro-inflammatory cytokines (13). Similarly, novel adjuvants like squalens or monophosphoryl lipid A (MPLA—a detoxified lipopolysaccharide) aim to enhance the innate immune response, but never reach the immunogenic potential of live attenuated vaccines (14). Adjuvants have been added to vaccines for more than 90 years and over the last decades, considerable progress has been made in understanding their mode of action and to improve safety (15). Besides the above mentioned aluminum salts, squalene and MPLA, oil emulsions, saponin, Toll-like receptor (TLR) agonists, enterotoxins, polysaccharides, and glycolipid adjuvants (16) are used, all of which stimulate the immune system as well.

Aluminum adjuvants have now been used for decades and lots of experience has been gained on its use, effectiveness, and safety and they still remain the most frequently used adjuvants. Their effects on the immune system comprise stimulation of macrophages and dendritic cells via PRRs, inflammasome activation, IL-1β release and activation of Th2 lymphocytes (15, 16). However, besides increased immunogenicity, aluminum adjuvants also increase reactogenicity and based on data from animal models and reports on narcolepsy, silicosis, Guillain-Barré-syndrome (GBS) and macrophagic myofasciitis, they are also discussed to induce autoimmunity (17). The second most commonly and long used adjuvants are oil emulsions. They have a strong reactogenic potential and can cause severe inflammatory local reactions such as ulceration and granulomas. The most well-known oil emulsion is complete Freund's adjuvant. However, due to its potent reactogenicity, it is not suitable for human use. A possible association between oil emulsions and autoimmunity disorders has been hypothesized from animal models. Oil emulsions are potent inducers of IL-1β and IL-17 (18, 19). IL-17 plays a major role in autoimmunity and MS and may trigger the migration of peripheral lymphocytes into the CNS across the BBB (20, 21). Frequently, a combination of adjuvants is used to increase immunogenicity of vaccines. AS03 is an adjuvant emulsion containing squalene, DL-α-tocopherol, and polysorbate 80. It is e.g., used for the pandemic swine flu vaccine Pandemrix® (15) or the FDA-licensed H5N1 monovalent influenza vaccine. In animal studies, autoimmunity was observed in connection with AS03 (22) and in humans, cases of narcolepsy have been reported (23). Oil emulsions are often combined with TLR agonists such as MPLA. Generally, TLR agonist adjuvants activate the inflammatory transcription factor NFκB AS04 is a combination of MPLA and aluminum salts and is used as adjuvant in vaccines against hepatitis B (Fendrix®) and HPV, as well as in the new recombinant vaccine against Herpes zoster. Most polysaccharide adjuvants activate NFκB to induce immune processes (e.g., dextran, zymosan) (24). However, delta-inulin for instance, a polysaccharide adjuvant used for Advax®, acts via NFκB-independent mechanisms to enhance humoral and cellular immune responses. Although the mechanisms are not yet fully understood, Advax® has so far not shown inflammatory side effects and has proven safety in hepatitis B vaccination and influenza (16).

After activation of the immune cascade and stimulation of dendritic cells, the latter increase their expression of MHC molecules and chemokine receptors such as CCR7 leading to their migration toward the draining lymph nodes in order to provide co-stimulatory signals for the differentiation of naïve T cells into immune effector cells (25). The activation of the immune cascade has various effects on T and B cells. In short, antigen-recognition by B cells leads to their activation and migration toward the T-B cell border of the lymph node, where they can subsequently receive additional stimuli by activated T helper (TH) cells. These signals include CD40 interaction, secretion of cytokines by TH1 or TH2 cells, and finally the transformation of B cells into plasma cells predominantly secreting low affinity antibodies (26). Later, the germinal center response contributes via affinity maturation (somatic hypermutation and affinity-based selection) and isotype switch to a sustained production of high affinity antibodies by predominantly plasma cells but also memory B cells. Basically, in the lymph nodes, numerous B cells with various affinity compete for the antigens presented by follicular dendritic cells. These antigens are processed and further presented via MHC II to follicular TH cells, which provide costimulatory signals (e.g., CD40, ICOS, and IL-21) leading to survival and further proliferation of B cells with highest affinity for the antigen (27).

In conclusion, vaccination-induced immune responses, including employed cell types and mediators, vary depending on the type of vaccine administration, kind of vaccine and choice of adjuvant. While antibodies will directly prevent and reduce infections, CD4^+^ and CD8^+^ T cells rather support the organism eventually reducing, controlling and clearing the pathogens. Antibodies bind to their antigen, neutralize pathogens, activate macrophages and neutrophils as well as the complement system, while CD4^+^ and CD8^+^ T cells secrete cytokines, perforins, and granzymes (9). The choice of adjuvant seems to be critical, since some may cause problems in autoimmune diseases. Thus, monitoring side effects regarding autoimmunity is essential.

### Vaccination and MS

In the early days of vaccine development, Louis Pasteur used nerve tissue of infected animals to obtain a rabies virus vaccine (28). Although saving countless lives it was recognized that active sensitization with neuronal tissue could occasionally lead to neuroparalytic autoimmune complications (29) with self-limiting autoimmune encephalomyelitis that fulfilled the pathological criteria of MS (29, 30). Advances in processing techniques and increasing insights in immunology led to modern vaccines devoid of neuronal tissue. MS is a chronic disease thought to be caused by immune-mediated mechanisms. Thus, immune responses caused by vaccinations will affect the immune system. However, their effects on immunology *per se*, but especially those in MS patients, are scarcely understood.

The same means by which infections can induce autoimmunity also apply for vaccination-induced immune activation. Possible structural similarities between microbial epitopes and epitopes of the CNS could lead to cross-reaction of antibodies via molecular mimicry as shown for streptococcal antibodies in heart tissue (31). Additionally, epitope spreading is a mechanism leading to a broadening of the immune response from the dominant epitope to cryptic (intramolecular) or neighboring molecules (intermolecular) resulting in an increased antibody repertoire and cellular response (32). Moreover, bystander activation, a process in which activated APCs stimulate autoreactive T cells, can occur (33). Bacterial and viral infections can trigger relapses and MRI activity in MS; vaccination has been proven to protect from or weaken infections, thus providing an “indirect” protection against MS disease activity (34).

Several reports on neurological disorders developing after immunization have been published including several cases on encephalomyelitic disorders (impaired consciousness, ataxia and optic neuritis) as well as demyelinating lesions in a patient with transverse myelitis after active immunization against influenza (35–38). Immunization against rubella was associated with diffuse myelitis and recurrent relapses with optic neuritis, paraparesis and impaired motor function (39, 40). Transverse myelitis (41) as well as optic neuritis (42, 43) were reported in patients vaccinated against measles, mumps and rubella. Further cases with symptoms suggestive for disseminated encephalitis were reported after vaccination against diphtheria-tetanus-poliomyelitis (DTP) (44) and after immunization against smallpox, rabies or typhus (45). Exacerbations of MS and demyelinating lesions were reported in MS patients and patients without a history of neurological conditions after immunization against hepatitis B (46). Similarly, Tourbah reported on 8 patients with demyelinating lesions and clinical symptoms after vaccination against hepatitis B (47).

In contrast to these case series, a case-control study (evidence class II) (48) including more than 440 patients with MS or optic neuritis and 950 controls without any underlying neuroimmunological disorder did not reveal an elevated risk for the development of MS or optic neuritis after immunization against hepatitis B, tetanus, influenza, measles/mumps/rubella, measles, or rubella (49). While Hernan came to same results for immunization against influenza or tetanus in a case-control study (evidence class II), active immunization against hepatitis B was reported to pose a higher risk for MS (50). The latter finding could, however, not be confirmed by Confavreux in a large case-crossover study. Additionally, no increased risk was seen for vaccination against tetanus and influenza as well (51). Similarly, other class II case-control studies did not report on an increased risk for MS after hepatitis B vaccination (52–54). An even decreased risk for MS was reported after tetanus immunization (8). In a large class I study, a patient register including 789,082 females vaccinated with the quadrivalent HPV vaccine was analyzed. Thereof, 4,322 patients with MS and 3,300 patients with other demyelinating disorders were studied and no increased risk for CNS manifestations was seen in this large cohort (55).

Miller et al. performed a prospective class II, randomized, double-blind, placebo-controlled study, which included 104 MS patients, who received either standard influenza vaccination or placebo. For a 6 months follow-up period, the occurrence of neurological symptoms or influenza was monitored and no differences were seen for relapse rates (56). A study by Langer-Gould reported on an increased risk for CNS demyelinating diseases within the first 30 days after vaccination. It was concluded that there is no increased risk for MS, but it seems that the transition from subclinical to overt autoimmunity in patients with existing disease is shortened (53).

Two major questions arise on the topic of “MS and vaccination”: (i) Can vaccines cause MS and (ii) can vaccines provoke or trigger relapses in patients with MS?

- Overall, the anecdotal reports associating MS onset and vaccination had limited reliability, lacked validity and could not be replicated in larger studies. Therefore, there is consensus that there is yet no evidence that MS can be caused by vaccines neither by inactivated nor by live vaccines (57).
- It is more difficult to assess the potency of vaccines to trigger relapses in MS patients. With respect to live vaccines it seems to be plausible that they may be able to provoke a deterioration of the disease, since they fulfill the criteria of an active infection with a replicative (although attenuated) organism. There is class IV evidence that at least the yellow fever (YF) 17D vaccine strain, which is derived from a natural occurring YF-virus and hasn't completely lost its neurotoxicity even after numerous passages, is able to provoke relapses in MS patients. However, it has to be kept in mind that the patient cohort had received immunomodulatory treatment and the sample size of this self-controlled case series study was rather small (58). The underlying potential immunologic mechanisms, which are responsible for this elevated relapse rate, are not understood yet and larger studies are necessary to confirm this association. Hypotheses may be generated based on observations after infections with helminths, mycobacteria and Epstein-Barr virus, or by the immunologic properties of this particular vaccine strain (59). Immunological analyzes showed that after immunization against YF, MS patients had a significantly increased MBP- and MOG-specific response shown by increased numbers of cells secreting interferon, IL-1α, IL-1β and tumor necrosis factor compared to unvaccinated MS patients or MS patients vaccinated against influenza (58).

Still, there is no evidence for other live vaccines such as MMR to deteriorate MS (57, 60). For inactivated vaccines, there is already more evidence available that an association between MS relapses and different kinds of vaccines does not exist (7). Even for vaccines, which were publicly accused to be associated with MS disease or relapse rate, like HPV or hepatitis B vaccines, there is no evidence to support any association between vaccination and clinical course of MS, as well as for vaccines containing inactivated neurotropic viruses like TBE (53, 61). It still remains unclear if inactivated vaccines may accelerate an upcoming relapse in patients with active MS by non-specific stimulation of cytokine production. However, data are scanty and most studies are underpowered leaving an uncertainty about very small risks (62).

### Adjuvants and MS

Besides effects of vaccines on induction and the disease course of MS, potential immunological effects of adjuvants have to be considered as well. Most experience on the possible induction of autoimmunity following administration of adjuvant-containing vaccines has been gained from animal models. However, results from experimental studies cannot be transferred to humans without reservation. First, the dose ratios tested in animal models are not the same as in humans and second, human immunology differs from animals. Indeed, oil emulsions, aluminum salts and squalene have shown severe side effects in animal models, while they are considered to be safe in humans (17).

An analysis performed by the European Medicines Agency (EMA) (63) investigated autoimmune disorders following vaccination against pandemic influenza A/H1N1 between October 2009 and December 2010 (64). Thirty percent of the 150 million doses of the distributed vaccines contained aluminum salts and squalene-based adjuvants. Overall, the study did not suggest a significant difference in the risk for autoimmune disorders for adjuvant and non-adjuvant vaccinations. ADEM was reported for 10 people (adjuvant vaccines: 7, non-adjuvant vaccines: 3), MS for 21 people (adjuvant vaccines: 20, non-adjuvant vaccines: 1), MS relapses for 24 patients (adjuvant vaccines: 21, non-adjuvant vaccines: 3), and one case of relapsing remitting MS was reported for adjuvant-containing vaccination (64). Statistical analysis revealed only a non-significantly increased risk for GBS (15). Also, a favorable benefit-risk profile of the vaccines was demonstrated (15, 65).

In conclusion, following the reports from literature, all of the EMA/FDA-approved vaccines (with exception for Yellow Fever) and adjuvants do not show a significantly increased risk for MS and ADEM. Constant improvement of basic immunological knowledge and technology will further improve the safety of adjuvants. Table 1 gives an overview of the recommendations of standard vaccinations in the general population and in MS patients.

**Table 1:** Overview of standard vaccination in the general population and MS patients.

**Vaccine**		**USA (CDC/ACIP)** **(66)**	**Germany (STIKO)** **(67)**	**Recommendation for multiple sclerosis**
Diphteria	Toxoid	All individuals	All individuals	Considered safe
Human papilloma virus	recombinant vaccine	All individuals 11-12a	All individuals 9-14a	Probably safe
Measles, mumps and rubella	live attenuated vaccine	All children and at-risk adults	Unprotected individuals and children exposed to kids	Probably safe, CAVE: Immunosuppression
Meningococcal A,C,W,Y	inactivated vaccine	At-risk individuals	At-risk individuals	Probably safe
Meningococcal B	recombinant vaccine	At-risk individuals	At-risk individuals	Probably safe
Pertussis	Toxoid	All individuals	All individuals	Probably safe
Pneumococcus	polysaccharide vaccine	All individuals > 65a and individuals at risk	All individuals > 60a and individuals at risk	Insufficient data
Tetanus	Toxoid	All individuals	All individuals	Considered safe
Varicella	live attenuated vaccine	Individuals lacking evidence of immunity	Seronegative individuals at risk	Probably safe, CAVE: Immunosuppression
Zoster	recombinant vaccine	All individuals > 50a	All individuals > 60a and individuals > 50 at risk	Insufficient data
Zoster	live attenuated vaccine	All individuals > 60a, recombinant preferred	Not recommended	Insufficient data, CAVE: Immunosuppression
Hepatitis B	recombinant vaccine	All children, individuals not at risk but who want protection from hepatitis B	All children, individuals at risk	Considered safe
Hepatitis A	inactivated vaccine	All children, individuals not at risk but who want protection from hepatitis A	All children, individuals at risk	Considered safe
Poliomyelitis	inactivated vaccine	All children	All children, individuals at risk	Considered safe
Haemophilus influenzae type b	Conjugate vaccine	All children, individuals at risk	All children, individuals at risk	Insufficient data
Tick-borne encephalitis	Inactivated vaccine	not available	Endemic areas and tick exposure	Probably safe
Yellow fever	live attenuated vaccine	endemic areas	endemic areas	Probably increased risk, CAVE: Immunosuppression
Rabies	inactivated vaccine	People at high risk of exposure	People at high risk of exposure	Considered safe
Influenza	inactivated vaccine	All individuals > 6 months	Individuals >65 years old, those with chronic diseases, and pregnant women	Considered safe
Influenza	live attenuated vaccine	Individuals 2a-49a with restrictions	Individuals w/ chronic disease 2-17a, inac. preferred	Not recommended

### Vaccination and ADEM

While there is a lot of literature on vaccination and risk for MS or MS relapses available, reports on vaccination and ADEM are scarce. Yet, ADEM has been discussed to be a sequelae of vaccinations (68) as well as to be preceded by infections. Several cases of ADEM have been reported to be timely related to vaccinations against rabies (69), HPV (70, 71), hepatitis A and B, diphtheria, tetanus and poliovirus (72), measles, rubella and booster immunization for Japanese encephalitis (73). ADEM has been reported following vaccination against influenza, including eight cases after vaccination against H1N1. Also, four ADEM cases after vaccination against YF can be found in literature (74, 75). Besides case reports, there have been some observational studies, albeit all having their limitations. In 26 out of 35 reported cases of ADEM, patients had infections or vaccinations prior to disease onset (76). Also, Pellegrino et al. concluded a possible relation between post-vaccination ADEM in children and adults. Four hundred four cases of ADEM were analyzed based on the data of the Vaccine Adverse Event Reporting System (VAERS) database and the EudraVigilance post-authorization module (EVPM) (77). About 60% of the cases occurred between 2 and 30 days after vaccination, most commonly against influenza and HPV. A case-control study on vaccination against hepatitis B, influenza, polio, diphtheria, pertussis, tetanus, measles, mumps, rubella, Japanese encephalitis, meningitis, hepatitis A, varicella and rabies did not reveal an increased risk for the onset of ADEM in the time spans of 0–30 days and 61–180 days after vaccination, but between 31 and 60 days (78). Based on these reports, the risk for ADEM after vaccination cannot be completely ruled out.

### Effective Vaccination in MS Treatment

Considerations on MS exacerbation and vaccination apply only for MS patients receiving no immunomodulatory/immunosuppressive treatment. If any kind of immunosuppression is used for MS therapy, this choice of treatment will dominate the decision whether to vaccinate or not (79). In recent years, consensus statements on vaccinations during immunosuppressive treatments were published by various national and international societies and expert panels (80–84). There is consensus that inactivated vaccines will do no harm (85) even in immunosuppressed patients. However, data on the efficacy of vaccinations in combination with the various available MS medications are missing. Thus, for patients either receiving more than one immunomodulatory treatment or having underlying immunomodulating condition, the outcome is difficult to predict (86). Therefore, the success of vaccination should be verified by antibody testing if a valid test is available.

Except for a few treatments, which only lead to mild immunosuppression, live vaccines are contraindicated under immunosuppressive treatment. In some situations, risks and benefits of a live vaccine have to be weighed against each other, e.g., in varicella zoster virus (VZV)-negative MS patients under fingolimod treatment, varicella vaccination may be considered, since severe complications from natural varicella infection may outweigh the risk from this live vaccine. However, recommendations vary between different institutions even within the same country (80, 82, 83). A recent case report on a lethal VZV infection in an immunocompromised patient after VZV live vaccination drives the discussion on this issue (87).

There is consensus about the timing of vaccination in patients, who will undergo immunosuppressive treatment: Vaccinations should be given well in advance to the start of treatment (at least 2 weeks for inactivated and ≥ 4 weeks for live vaccines) and should be distinguished between primo-vaccinations and boosters. Importantly, the refractory period after immunosuppression has to be considered as well, which may be up to 1 year depending on the type of medication (e.g., rituximab or alemtuzumab) (81). Vaccines will have various effects on the immune system, which greatly depend on the cell types typically engaged by the respective vaccines. The impact of immunosuppression on the various cell types (and possible mitigation of effects) should be taken into consideration. Protective efficacy is mostly mediated by antibodies for the following vaccines: cholera, diphtheria toxoid, hepatitis A and B, haemophilus influenzae type b, influenza, Japanese encephalitis, meningococcal PS and conjugates, papillomavirus, pneumococcal PS and conjugates, polio (Sabin and Salk), rabies, rotavirus, rubella, tetanus toxoid, typhoid PS, and YF. Effects are solely born by T cells for tuberculosis (BCG), or by a combination of antibodies and T cells for measles and intranasal influenza vaccination. Besides antibody-mediated protection, effects of T cells are discussed for pertussis (9).

For patients receiving immunosuppressive treatment, vaccination control should be performed. For diphtheria, TBE (with caution), hepatitis A, B, haemophilus influenzae type b, measles, mumps, pneumococcus, polio, rubella, tetanus, rabies and varicella, standards are available and recommended to be tested. In general, to increase the validity of vaccination control, titers should be assessed in paired samples (before and after immunization) via the same method and at high-quality standards (81). In general, patients should have received their recommended standard vaccines according to their region-specific vaccine guidelines. Before certain immunosuppressive treatments are initiated, it is mandatory to exclude former infections and if necessary, vaccination should be considered according to the regulatory agencies. Table 2 provides an overview on necessary vaccinations according to FDA/EMA guidelines (extended vaccination reflects the authors' suggestion). For many immunotherapies, a prior exclusion of an ongoing VZV infection is required and vaccination should be offered to those, who haven't gained any immunity yet. Additionally, VZV-seropositive patients undergoing immunotherapy should be offered vaccination as well to prevent zoster reactivation and late effects. Recently, a non-live subunit vaccine has been authorized for VZV-seropositive patients. It possesses a better risk-benefit profile compared to the live vaccine and has already been approved by many countries (88).

**Table 2:** Recommended vaccination in MS patients in dependency of treatment.

	**FDA/EMA vaccination**	**FDA/EMA screening**	**Extended vaccination**
GLAT			
IFN beta			
Cladribin	VZV	Screen for HBV, HCV	
Teriflunomid			
Fingolimod	VZV		HBV, HPV
DMF			
Rituximab	n.a.	n.a.	HBV, Pneumococcal
Ocrelizumab		Screen for HBV, HCV	HBV, Pneumococcal
Natalizumab			VZV
Alemtuzumab	VZV	Screen for HBV, HCV	HBV, Influenza, HPV and Pneumococcal

*GLAT, glatiramer acetate; IFN beta, interferon beta; DMF, dimethyl fumarate; HBV, hepatitis B; HCV, hepatitis C; VZV, varicella-zoster virus; HPV, human papillomavirus; n.a., not applicable*.

Additionally, it should be considered to offer patients with upcoming fingolimod or alemtuzumab treatment the option of vaccination against HPV, as post-market surveillance showed increased reports of warts and cervical dysplasia due to these two MS therapies [EMA; (89)]. Furthermore, pneumococcal vaccine might be considered in patients receiving B cell-depleting therapies, as severe respiratory infections during Phase III studies were seen (90, 91).

## Discussion

Vaccine hesitancy is a major problem nowadays. The usefulness of active immunization is undisputed and has saved numerous lives. However, fear of possible, but also often unconfirmed, side effects has fostered this anti-vaccine sentiment. This has led to a recent outbreak of measles (2) and curiously some viruses and disorders, which have been assumed to be eradicated, seem to become a hot topic for Western health systems again.

Indeed, side effects upon vaccination may occur in rare cases, however, the benefits for individual people as well as the whole population will generally outweigh adverse effects. Vaccine hesitancy results in a twofold problem: (1) The missing protection for the unvaccinated people themselves but also (2) a risk for people, who are not able to get vaccinated. The missing herd immunity poses a major problem for a group of patients with fragile health. For MS patients receiving immunosuppressive treatment, an acute infection can have dangerous sequelae. Thus, if possible, MS patients should be vaccinated beforehand. The possible benefits outweigh—dependent on the individual case—the possible risks.

An additional perspective raises the possibility of vaccination against MS. Indeed, early approaches exploring vaccination with synthetic peptides in experimental animal models were successful, but translation into clinical treatment was so far unsatisfying (92–94).

Interestingly, it was recently shown that an anti-typhus vaccination (Typhim vaccine) might have the potential to ameliorate the disease course of MS by targeting prohibitins on TH17 cells. Tested in an experimental MS model it led to decreased levels of IL17 and increased numbers of FOXp3^+^ regulatory T cells (95). Further investigations are needed before studies should investigate treatment options for MS patients. Still, it is a good example, how immunology of vaccination might overlap with and modulate the immunology of MS.

## Conclusion

Theoretically, an increased immune response against different types of vaccines, such as live attenuated viruses, inactive attenuated viruses, or portions of bacteria and viruses, could trigger increased immune response to self-antigens (45, 58, 96), but an increased risk for MS itself or increased relapse rates after vaccination have not been show (with exception for YF) in case-control studies (7). There is, however, evidence that infections can trigger relapses in MS (96–104), which is why vaccination of MS patients should be pursued in order to reduce the risk of infections. To assure the best vaccination success, immunization and immunosuppressive treatments have to be well timed.

## Author Contributions

All authors listed have made a substantial, direct and intellectual contribution to the work, and approved it for publication.

### Conflict of Interest Statement

The authors declare that the research was conducted in the absence of any commercial or financial relationships that could be construed as a potential conflict of interest.
